# Application of herbal traditional Chinese medicine in the treatment of lupus nephritis

**DOI:** 10.3389/fphar.2022.981063

**Published:** 2022-11-24

**Authors:** Liu Liu, Lei Zhang, Ming Li

**Affiliations:** The First Affiliated Hospital of Anhui University of Traditional Chinese Medicine, Hefei, China

**Keywords:** lupus nephritis (LN), traditional Chinese medicine, inflammation, therapy, kidney injury

## Abstract

Lupus nephritis (LN) is a secondary renal disease caused by systemic lupus erythematosus affecting the kidneys. It is one of the main causes of end-stage renal disease and a serious risk factor for early mortality and disability of systemic lupus erythematosus patients. Existing LN treatment is mainly based on hormones, cytotoxic drugs, and biological agents. Nevertheless, the prognosis of LN patients remains poor because of frequent recurrence and exacerbation of adverse drug reactions. Hence, LN is still the most important cause of end-stage renal disease. In recent years, traditional Chinese medicine (TCM) has attracted increasing attention because of encouraging evidence that it alleviates LN and the well-described mechanisms underlying renal injury. TCM has therapeutic benefits for treating LN patients. This review article elucidates TCM preparations, TCM monomers, and herbal or natural extraction for LN treatment to provide effective supplementary evidence for promoting the development of TCM treatment for LN and reference for future research and clinical practice.

## Introduction

Systemic lupus erythematosus (SLE) is a chronic autoimmune disease caused by the abnormal activation of lymphocytes leading to the inappropriate production of large amounts of autoantibodies, which affect multiple systems and organs (Durcan et al., 2019). Inflammatory nephropathy caused by the immune complex deposition involving the kidneys has a long course and is prone to recurring attacks. It can cause serious kidney injury if poorly managed. More than 1/4 of SLE patients have kidney injury at the onset, and 90% of them have pathological manifestations of renal involvement during the onset. The incidence of end-stage renal disease is approximately 4.3% and is a serious risk factor for early mortality and disability of SLE patients. It is still one of the three major causes of mortality in SLE patients (Hanly et al., 2016; Ocampo-Piraquive et al., 2018; Raimbourg and Daugas, 2019; Anders et al., 2020). Lupus nephritis (LN) is the most common and critical visceral complication of SLE, the main cause of mortality in SLE patients, and still the principal cause of end-stage renal disease (Lee et al., 2016; Gasparotto et al., 2020; Mahajan et al., 2020). In modern medicine, hormones, cytotoxic drugs, and biological agents have often been used in clinical practice to control LN, improve the prognosis of the patients, and reduce the toxicity and adverse effects of drugs to continuously improve the therapeutic efficacy of LN drugs.

The pathogenesis of LN has not been fully clarified yet, existing reports have shown that immunomodulatory imbalance and inflammation are important in LN pathogenesis (Frangou et al., 2020), which is mainly considered to be related to immune complex deposition (IC), complement system abnormality, immune cell abnormality and cytokine change, gene expression (epigenetic modification) abnormality, virus immunity, and viral immunity. The main pathogenesis of LN is the activation of complement system caused by the deposition of immune complexes in the kidney. Typical lupus glomerulonephritis is driven by immune complex mediated inflammation. The complement cascade reaction in renal tissue is caused by the deposition of immune complex, which promotes the proliferation and activation of glomerular mesangial cells and releases a variety of inflammatory factors (Rekvig, 2019; Chang et al., 2021). The autoantibodies of the kidney itself (antibody GBM antibody) form immune complexes *in situ* in the kidney, which also cause kidney damage. In addition, abnormal immune cells and changes in cytokines are also important aspects of its pathogenesis (Kant et al., 2022). Neutrophil extracellular traps (NETS), as immune cells, play an increasingly important role in the pathogenesis of LN. Neutrophils induce plasma cell like dendritic cells to produce IFN-α, which is involved in the injury of endothelial cells (Nishi and Mayadas, 2019; Bruschi et al., 2021). CD4+T cells are auxiliary signals for B cell differentiation, which induce B cells to secrete a large number of autoantibodies to form immune complexes. Nucleic acid of immune complex initiates renal inflammation through TLR in macrophages and dendritic cells (Choi and Morel, 2017; Obrișcă et al., 2021). Epigenetics (DNA methylation, abnormal expression of mi RNA, histone modifications, etc.) are also involved in the development of several autoimmune diseases, including LN, and abnormal epigenetic modifications directly lead to abnormal proliferation of renal cells, renal fibrosis and inflammation in LN (Mei et al., 2022; Xu et al., 2022). Hence, the development of new and effective therapeutic regimens to regulate immune function and control inflammatory response is of great significance for LN treatment and the improvement of disease conditions and prognosis.

Because of the complexity, cost, and poor efficacy of LN treatments, supplementary and complementary treatment options are becoming increasingly attractive (Balkrishna et al., 2020; Lu et al., 2021). Chinese herbal medicine, which has been practiced for thousands of years, remains one of the leading treatments in China and East Asia. In the 21st century, it has rapidly spread worldwide. After thousands of years of practice and research, the potential therapeutic benefit of traditional Chinese herbal medicine for LN patients has gradually been recognized. Clinical trials and observational studies have provided encouraging evidence that Chinese herbal medicine is beneficial to LN patients. Recent studies have reported that many TCM Preparations and Monomers and extracts of these drugs have significant efficacy in the treatment of immune diseases, such as the classic formula Huang-Lian Jie-Du decoction (HLJDD), which has unique clinical efficacy in the treatment of immune diseases such as lupus nephritis, rheumatoid arthritis, and ulcerative colitis. Nie et al. (2016) found that HLJDD significantly inhibited STAT3 phosphorylation and thus exerted renoprotective effects in the treatment of lupus nephritis (LN) mice; Li et al. (2021) found that the potential mechanism of HLJDD in the treatment of RA may be attributed to the inhibition of immune inflammatory response, reduction of chemokine release, and attenuating the destruction of extracellular matrix (ECM) in the synovial compartment; Yuan et al. (2020) suggesting that HLJDD improves acute ulcerative colitis in mice by modulating NF-κB and Nrf2 signaling pathways and enhancing intestinal barrier function.

TCM monomers such as Tripterygium wilfordii also have significant anti-inflammatory and immunosuppressive properties (Song et al., 2020) and are useful in the treatment of systemic lupus erythematosus, rheumatoid arthritis, ankylosing spondylitis, systemic sclerosis-associated interstitial lung disease (SSc ILD), and chronic urticaria have definite clinical efficacy (Liu et al., 2018; Chen et al., 2020; Yang et al., 2020; Zhang et al., 2021a; Zhang et al., 2021b); Triptolide is an extract of Tripterygium wilfordii, which exerts its anti-inflammatory, immunosuppressive and anti-tumor activities by regulating cellular autophagy, apoptosis, antioxidant and other multiple pathways, and is now widely used in the clinical treatment of immune diseases, tumors, kidney diseases and other immune diseases and has become a popular research direction (Qin et al., 2018; Wei et al., 2019; Ren et al., 2020; Yu et al., 2021a). Recent studies have reported that many compound formulas and herbal monomers and extracts of these drugs have significant efficacy in the treatment of immune diseases, such as the classical formula Huang-Lian Jie-Du decoction have unique clinical efficacy in the treatment of immune diseases such as lupus nephritis1, rheumatoid arthritis2 and ulcerative colitis2. TCM Monomers such as Tripterygium wilfordii have definite clinical efficacy in the treatment of systemic lupus erythematosus3, rheumatoid arthritis4, ankylosing spondylitis5, systemic sclerosis-associated interstitial lung disease (SSc-ILD)6, and chronic urticaria7; Triptolide, an extract of Tripterygium wilfordii, is currently used in the treatment of lupus nephritis1, rheumatoid arthritis2, and ulcerative colitis2 because of its immunosuppressive effect. It has been widely used in the clinical treatment of immune diseases, tumors, kidney diseases and other immune diseases due to its good immunosuppressive effect, and has become a popular research direction. The combination therapy of traditional Chinese medicine (TCM), steroids and immunosuppressants based on the principle of combination of disease and syndrome is effective in reducing adverse effects and recurrence rates and increasing the therapeutic efficacy in LN (Choi et al., 2018; Yu et al., 2021a). Significant benefits of Chinese herbal medicine that have been observed and reported include improvement of symptoms, reduction of antibody and proteinuria levels, improvement of kidney injury, reduction in the dose and toxicity of hormones used, and prevention of disease flares (Wu et al., 2018; Du et al., 2022; Liu et al., 2022). This review summarized the therapeutic effects and mechanisms of various traditional Chinese medicines (preparations, monomers and extracts) on lupus nephritis (Table 1).

**TABLE 1:** Herbal traditional Chinese medicine for the treatment of lupus nephritis.

Names	Origins	Functions	Effects
Liuwei Dihuang pills	Radix Rehmanniae praeparata, Rhizoma Dioscoreae, Fructus Corni, Cortex Moutan, Poria, and Rhizoma Alismatis	Reduces the chemokine expression of fractalkine	Preventing the recurrence Chang et al. (2017)
Zhibai Dihuang pills	Liuwei Dihuang pills, Rhizoma Anemarrhenae and Phellodendron chine	Antioxidant, anti-inflammatory, and immunomodulatory properties against epithelial damage	Improving renal function Zhang et al. (2014)
Ba-Wei-Dihuang pills	Liuwei Dihuang pills, cinnamon, and monkshood plant	Tonifying yang and nourishing the kidneys	Relieves nephritis and reduces proteinuria and immune complex precipitation Furuya et al. (2001)
Huang-Lian Jie-Du decoction	Rhizoma Coptidis, Cortex Phellodendri, Radix Scutellariae	Antibacterial, anti-inflammatory, antioxidant	Lowering renal immune complex C3 deposition in the LN mouse model Lai et al. (2019)
Zhenwu decoction	Poria, Chinese herbaceous peony, ginger, Fuzi, and Baizhu	Controlling lupus activity	Improving renal function, and accelerating disease remission Duan et al. (2021)
Tripterygium wilfordii	Thunder god vine, is from the order Celastrales	Promotes dryness and blood circulation to remove meridian obstruction	Have a therapeutic effect, some of its active components may also cause adverse effects Yingyan et al. (2022)
Paeonia	The dried root of red peony (Paeonia anomala of family Ranunculaceae) or Paeonia veitchii	Clearing heat, cooling blood, dispelling stasis, and relieving pain	Prevents the adhesion of inflammatory cells Ding et al. (2009)
Cordyceps sinensis	A unique leaf-like fungus that grows on caterpillars	Invigorating the kidneys, improving lung function, and enhancing the original qi	Have a potential effect on improving the immune function Barido et al. (2020), Li et al. (2020)
Artemisia	A sesquiterpene lactone drug with a peroxide group	Anti-inflammatory and immunomodulatory	Reduces tissue damage caused by humoral and cellular immunity, and enhances immune tolerance Aldieri et al. (2003), Wu et al. (2016)
Curcumin	A polyphenolic monomer	Anti-inflammatory, antioxidation, antiproliferation, and immunomodulation	Protective effect against kidney injury Hou et al. (2019), Liu et al. (2019), and Fanouriakis et al. (2020)
Loquat leaf and Osmanthus extracts	Three types of loquat leaf methyl coralline, ursodiol, oleanolic acid, and acetyl oxyl-oleanolic acid, isolated from Osmanthus	Anti-inflammatory, produce inflammatory cytokines	Improving the pathological damage and reducing the severity of renal damage Zhou et al. (2020)
Realgar	An arsenic tetrasulfide compound	Anti-inflammatory	One of the therapeutic targets of LN Xu et al. (2019)

## Traditional Chinese medicine preparations in lupus nephritis

### Liuwei Dihuang pills for lupus nephritis

China is well known for its use of traditional medicine. The Liuwei Dihuang pill is one of the most popular Chinese herbal medicines with a significant curative effect on chronic kidney disease, containing six medicinal compounds: Radix Rehmanniae praeparata, Rhizoma Dioscoreae, Fructus Corni, Cortex Moutan, Poria, and Rhizoma Alismatis. It was first reported in the ancient Chinese literature *Tips for the Treatment of Pediatric Diseases* in the Northern Song dynasty (960–1127 AD) (Li and Zhang, 2013; He et al., 2019). According to the TCM theory, Liuwei Dihuang pills have the functions of nourishing *yin* and tonifying the kidneys. In modern research, Liuwei Dihuang pills boost the immune system, improve renal function, and promote metabolism (Liu et al., 2020; Qiu et al., 2020; Hou et al., 2021). Relevant clinical studies have shown that Liuwei Dihuang pills in combination with hormone therapy in the treatment of LN reduce receptor expression, consequently reducing the advanced glycation end-product levels to avoid further exacerbation of tissue immune responses. Reduction of monocyte chemoattractant protein 1 expression inhibits the release of inflammatory mediators, such as interleukin (IL)-1 and IL-6, and reduces the chemokine expression of fractalkine to control inflammatory exudation (Chang et al., 2017), thereby significantly improving the efficacy of hormone therapy, reducing the adverse reactions of hormone therapy drugs, and preventing the recurrence of LN.

### Zhibai Dihuang pills for lupus nephritis

Zhibai Dihuang pills are composed of Liuwei Dihuang pills, and *Rhizoma Anemarrhenae* and *Phellodendron chine*, which are pungent cold herbs promoting fluid production, nourishing *yin*, removing “fire” toxin, tonifying deficiency, and replenishing *qi*. Zhibai Dihuang pills have antioxidant, anti-inflammatory, and immunomodulatory properties against epithelial damage, which improve nephritis. The addition of *Phellodendron chine* inhibits mRNA levels of anti-inflammatory cytokines, including tumor necrosis factor-alpha (TNF-α), IL-1β, IL-6, and cyclooxygenase 2 (Cheng et al., 2019). *Rhizoma Anemarrhenae* regulates AMP-activated protein kinase K activation for anti-inflammation and immunomodulation. Furthermore, the combination of *Rhizoma Anemarrhenae* and *Phellodendron chine* exerts an anti-inflammatory effect by regulating the Akt/mTOR/FoxO signaling pathway to enhance the anti-inflammatory activity of LN (Zhang et al., 2014). These effects inhibit symptoms of inflammation, and regulate cellular and humoral immunity, assisting in the repair of glomerular basement membrane damage, reducing thylakoid immunoglobulin G and C3 deposition, and improving renal function.

### Ba-Wei-Dihuang pills for lupus nephritis

Ba-Wei-Dihuang pills are composed of Liuwei Dihuang pills, cinnamon, and monkshood plant (*Aconitum*), which has the functions of tonifying *yang* and nourishing the kidneys based on the TCM theory. They are used to treat various aging-related diseases, including low back pain, paresthesia, edema, urinary hesitancy, and blurred vision. Recent studies have shown that Ba-Wei-Dihuang pills ameliorate autoimmune diseases, such as LN, through various mechanisms. Administration of Ba-Wei-Dihuang significantly relieves nephritis and reduces proteinuria and immune complex precipitation. The specific mechanism is mainly by improving the imbalance of T-helper 1 (Th1) dominance in the body. Ba-Wei-Dihuang pills reduce serum double-strand (ds)DNA antibody levels and significantly inhibit the production of antigen-specific interferon (IFN)-γ, thereby further inhibiting the production of IL-12 (Furuya et al., 2001). Moreover, a previous study showed that Ba-Wei-Dihuang pills limited the expression of the IL-18 receptor complex by reducing the production of IL-4 by natural killer T cells, thereby reducing the hyperresponsiveness of cells to IL-18 (Furuya et al., 2003), normalizing the Th1 imbalance, and controlling the autoimmune diseases in mice.

### Huang-Lian Jie-Du decoction

The earliest report of Huang-Lian Jie-Du decoction (HLJDD) was in *Zhouhoubeijifang* (*Handbook of Prescriptions for Emergencies*), and the name first appeared in *Waitai Miyao* (*Medical Secrets of an Official*). HLJDD is composed of four herbs, including *Coptis chinensis, Phellodendron chinensis and scutellaria chinensis, and gardeniae fructus*, in a 3:2:2:3 ratio. It is a Chinese medicine formula for purging “fire” and removing toxins and has been widely used in TCM treatment for cardiovascular and cerebrovascular diseases, inflammation, senile dementia, and diabetes. It has a wide range of pharmacological activities (e.g., antibacterial, anti-inflammatory, antioxidant, and neuroprotective activities) and multiple functions (e.g., antiendotoxin, anticoagulation, immunomodulatory, anti-inflammatory, and antiviral functions). It effectively improves the hypoxia–ischemia resistance of tissues and organs and endothelium-dependent vasodilation, reduces creatinine levels, and inhibits the expression of angiotensin II and inflammatory cytokines, thereby effectively protecting the renal function of patients (Lu et al., 2011; Yi et al., 2012; Khan et al., 2016; Kumar et al., 2016; Li et al., 2016; Nie et al., 2016). In LN, experiments confirmed that HLJDD reduced urine protein concentration and creatinine concentration, alleviated renal lesions, improved renal function, and decreased the mortality rate of an LN mouse model. These effects were achieved by inhibiting the activation and phosphorylation of p-STAT3, thereby inhibiting the JAK/STAT signaling pathway to prevent the release of inflammatory cytokines (namely, IL-6, IL-10, and IFN-γ), reducing autoimmune activity, inhibiting renal macrophage infiltration, and lowering renal immune complex C3 deposition in the LN mouse model (Lai et al., 2019).

### Zhenwu decoction

Zhenwu decoction was first reported in *Shanghan Lun* (*Treatise on Febrile Diseases Caused by Cold*). It is a famous TCM formula for warming *yang* and excreting water. It consists of Poria, Chinese herbaceous peony, ginger, *Fuzi* (*Radix Aconiti Carmichaeli*), and *Baizhu* (*Atractylodes macrocephala Koidz*). It is clinically used for the treatment of the symptoms of *yang* deficiency in the spleen and kidneys, such as urinary hesitancy; heavy or swollen limbs; pale, enlarged, and white tongue; and sunken pulse. The pathogenesis of LN involves immune complex deposition in the glomerulus, including the circulating immune complexes and *in situ* immune complexes, both of which activate the complement system, cause inflammatory cells (e.g., neutrophils and platelets) to aggregate, produce a series of reactions that damage the kidneys, and eventually cause LN. In clinical research, Zhenwu decoction has been reported to have an overall curative effect on LN patients, relieving clinical symptoms, controlling lupus activity, improving renal function, and accelerating disease remission (Duan et al., 2021). Thus, this TCM is worthy of clinical application.

**FIGURE 1:**
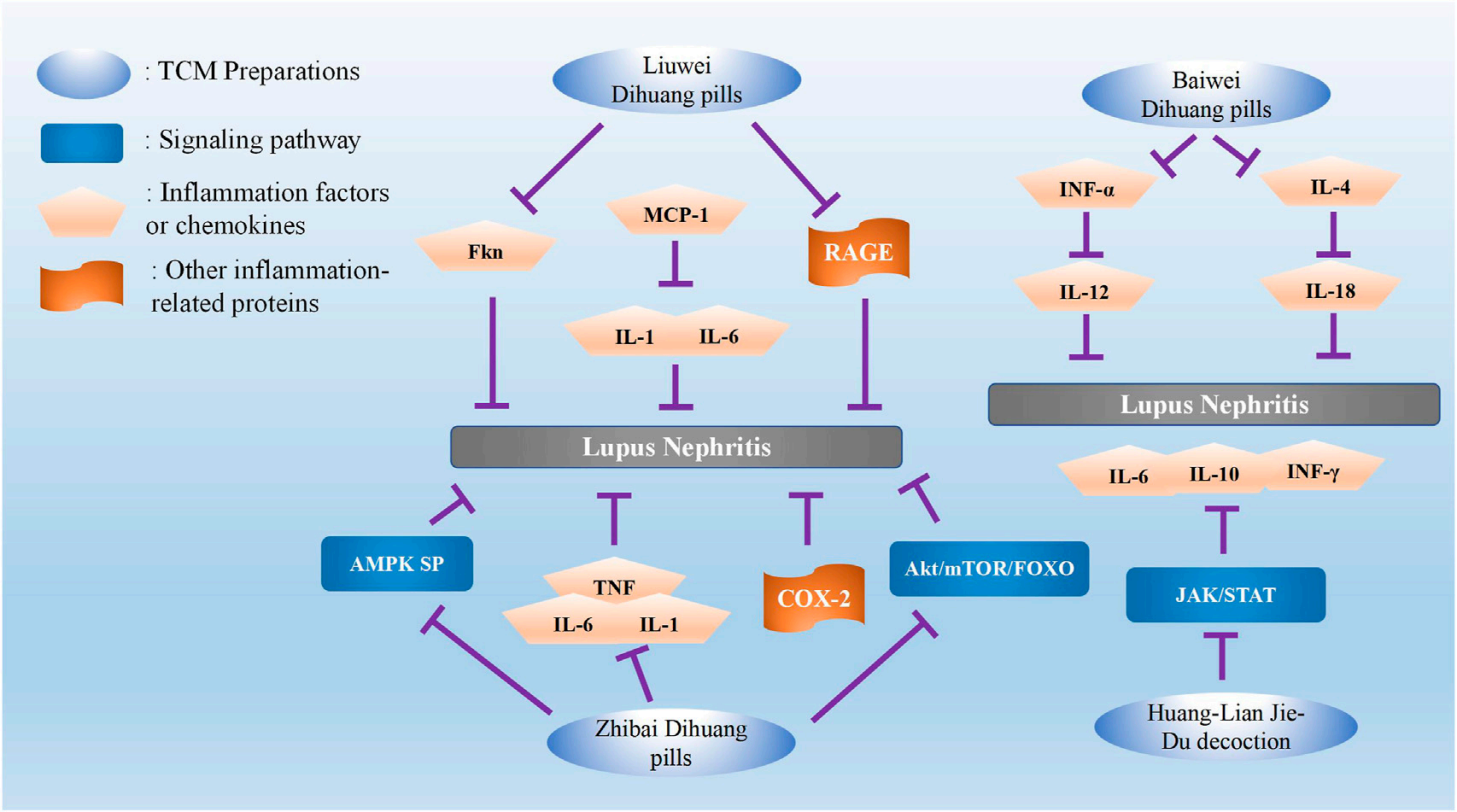
Summary of the role of TCM Preparations in the treatment of lupus nephritis.

In summary, previous studies have confirmed that the aforementioned TCM formulae for nourishing kidney *yin*, invigorating and strengthening kidney *yang*, clearing heat, and eliminating dampness and heat can be combined with Western medicine as faster, better, and safer treatments for LN. Furthermore, many studies have reiterated the effectiveness and applicability of the aforementioned TCM formulae at the molecular level, further highlighting the key roles of TCM treatment. However, TCM prescriptions mainly activate blood, clear heat, and detoxify, and the aforementioned TCM formulae are based on the disorder of the autoimmune system in LN. The molecular mechanisms of the TCM formulae are resolving homeostatic disorders and imbalance and restoring normal homeostasis making them the top choice in the clinical treatment of LN (Figure 1).

## Traditional Chinese medicine monomers in lupus nephritis

### Tripterygium wilfordii

*T. wilfordii*, also known as thunder god vine, is from the order Celastrales. Its dried root has a bitter and acrid taste. It promotes dryness and blood circulation to remove meridian obstruction, and it is used as a treatment for LN, rheumatism, and kidney diseases (Feng et al., 2018). Modern pharmacological research has shown that *T. wilfordii* has anti-inflammatory, analgesic, and immunosuppressive effects and has therapeutic effects similar to those of glucocorticoids. In China, it has become a commonly used drug for the treatment of LN. However, *T. wilfordii* preparations also cause kidney-related adverse reactions, such as renal failure, renal insufficiency, hematuria, oliguria, and increased creatinine level (Yingyan et al., 2022). Therefore, more caution should be exercised when choosing *T. wilfordii* as an LN treatment. Studies have shown that the therapeutic effect of *T. wilfordii* on LN is mainly manifested in the inhibition of immunity and inflammation, renal intestinal fibrosis, and vascular damage (Lesiak et al., 2010; Hongqin et al., 2011; Qu et al., 2014; Lu et al., 2015; Lu et al., 2016; Drehmer et al., 2017; Lee et al., 2019; Yingyan et al., 2022). Celastrol (tripterine), a compound isolated from the dried root of *T. wilfordii*, inhibits the expression of tissue inhibitor of metalloproteinase 1 in the kidneys and has a significant effect on delaying glomerulosclerosis in a lupus rodent model (Xiang et al., 2022). Estrogen receptor 1 encodes estrogen receptor alpha, and its expression is positively correlated with antinuclear antibody and antireceptor-associated protein antibody and the severity of clinical symptoms of lupus erythematosus (Wang et al., 2020a). Triptolide, a compound isolated from *T. wilfordii*, upregulates the expression of caspase-3 and caspase-9 (Zubair and Frieri, 2013) and may worsen the symptoms of proteinuria. Vascular endothelial growth factor A (VEGFA) is a core target in the protein–protein interaction network, and its receptor, Fms related receptor tyrosine kinase 1, has the function of protecting the integrity of endothelial cells and the vascular system and the glomerular filtration barrier in an inflammatory environment (Tan et al., 2020). In LN, VEGFA synthesis is decreased and the serum receptor Fms related receptor tyrosine kinase 1 is increased, resulting in insufficient VEGFA in renal tissues, which leads to fibrosis and proteinuria. Most of the components of *T. wilfordii* have a therapeutic effect on LN, but some of its active components may also cause adverse effects such as podocyte apoptosis and accelerated renal fibrosis.

### Paeonia

The dried root of red peony (*Paeonia anomala* of family Ranunculaceae) or *Paeonia veitchii* Lynch is a commonly used TCM in clinical practice. According to the TCM theory, it has the functions of clearing heat, cooling blood, dispelling stasis, and relieving pain (Liang et al., 2021). Modern experimental research also provides pharmacological evidence that red and white peony (*Paeonia lactiflora*) dispel wind dampness and eliminate blood impediments. The main chemical active ingredient extracted from peony roots is total glucosides of peony (TGP) (Wang et al., 2020b; Jiang et al., 2020). Peony roots also contain tannins, flavonoids, and volatile oils. White and red peony roots have higher contents of monoterpene glycosides, such as paeoniflorin, albiflorin, oxypaeoniflorin, and benzoyloxy paeoniflorin. They have anti-inflammatory, immunomodulatory, analgesic, sedative, antistress, antiulcer, and hepatoprotective functions and fewer adverse effects. *Paeonia* has been extensively used in TCM treatment of rheumatoid arthritis and rheumatic diseases, such as arthritis, SLE, and ankylosing spondylitis.

Although the molecular mechanism of TGP has not yet been investigated, LN, which is the most serious complication of SLE, may have similar mechanisms to SLE. TGP mainly downregulates the expression of IL-8, TNF-α, and IFN-α in SLE patients and, therefore, has a protective effect against SLE. It is speculated that LN treatment may also be related to its ability to inhibit IL-1, IL-8, TNF-α, IFN-α, prostaglandin E_2_, and other cytokines, which, however, still need further experimental verification. Animal studies have shown that in a mouse model, red peony reduces the expression of intercellular adhesion molecule-1, vascular cell adhesion molecule-1, and platelet endothelial cell adhesion molecule-1; prevents the adhesion of inflammatory cells; and improves the effect of the LN (Ding et al., 2009). Nevertheless, a study on the specific mechanism and further clinical trials are required to verify the results.

### Cordyceps sinensis

*Cordyceps sinensis* is a unique leaf-like fungus that grows on caterpillars and is considered a tonic in TCM to treat various diseases. In the TCM theory, *C. sinensis* has the functions of invigorating the kidneys, improving lung function, and enhancing the original *qi*. It has been suggested that *C. sinensis* regulates the immune system bidirectionally, reduces tubulointerstitial damage, inhibits renal fibrosis, and improves renal functions (Barido et al., 2020; Li et al., 2020). In six randomized controlled trials with treatment duration of 2–12 months, the effects of *C. sinensis* were tested on a total of 507 LN patients (Li et al., 2006; Davidson et al., 2019; Ren et al., 2019; Yu et al., 2021b). In the Western medicine control group, the LN patients received glucocorticoid (methylprednisolone or prednisone) and immunosuppressive agents (cyclophosphamide or tacrolimus). In the *C. sinensis* plus Western medicine treatment group, the LN patients received *C. sinensis* preparations (1 or 1.65 g of *C. sinensis*, three times daily) and equal doses of Western medicine as in the control group. Compared with the Western medicine control group, the group that received combination therapy of *C. sinensis* preparations and Western medicine had significantly improved clinical symptoms and decreased disease activity scores of LN, 24-h urine protein, anti-dsDNA antibody, and serum creatinine levels. Furthermore, the infection rate and adverse reaction rate in the *C. sinensis* plus Western medicine treatment group were significantly lower than in the Western medicine control group. These results suggested that *C. sinensis* may have a potential effect on improving the immune function of LN patients, and the combined application of *C. sinensis* and Western medicine had a clinical curative effect on LN.

## Herbal or natural extracts

### Artemisia

Artemisinin is a sesquiterpene lactone drug with a peroxide group. It is extracted from the leaves of *Artemisia annua*. The annua has been used to treat malaria in China for over two thousand years. Its derivatives are artemether, arteether, artesunate, and dihydroartemisinin. *A. annua*, artemisinin, and its derivatives are a great potential source of TCMs. These newly identified artemisinin derivatives have significant immunosuppressive activity and therapeutic safety, acting in all stages of innate and acquired immunity to exert anti-inflammatory and immunomodulatory effects. It also inhibits helper T cells, promotes the proliferation of regulatory T cells, inhibits mature B cells, reduces tissue damage caused by humoral and cellular immunity, and enhances immune tolerance (Okorji et al., 2016; Wu et al., 2016). In recent years, research teams in China have designed new artemisinin derivatives with lower toxicity, high biological activity, and immunosuppressive activity, such as SM934, SM905, SM735, and SM933. Animal experiments on SM934 suggest that SM934 promotes the expression of IL-10, inhibits pathological T cells (e.g., Th1 and Th17), and increases the number of resting B cells while reducing the number of activated B cells and plasma cells. As a candidate drug for SLE treatment, SM934 has been approved by the U.S. Food and Drug Administration for clinical studies in the treatment of lupus (Hou et al., 2011; Xiao et al., 2022).

### Curcumin

Curcumin (Cur) is a polyphenolic monomer used in TCM treatment. It has various pharmacological effects, such as anti-inflammatory, antioxidation, antiproliferation, and immunomodulation. It has been extensively used in the treatment of various diseases and has been confirmed to have a certain protective effect against kidney injury (Hou et al., 2019; Liu et al., 2019; Fanouriakis et al., 2020).

Fan et al. (Ding et al., 2011) showed that Cur inhibited Akt phosphorylation and upregulated APPL1 expression, thereby protecting the acute kidney injury caused by ischemia–reperfusion. In MRL/lpr mice, Cur has a significant nephroprotective effect, and its mechanism of action may be related to the inhibition of the nuclear factor kappa B signaling pathway and the activation of NLRP3 inflammasome, (Zhang et al., 2019; Zhang and Wei, 2020).

### Loquat leaf and *Osmanthus* extracts

Loquat leaf is a type of Chinese medicine with an anti-inflammatory effect. Many studies have shown that Th17 cells play a crucial role in mediating the pathological deterioration of SLE. Th17 cells have also been shown to play a key role in the pathogenesis of various autoimmune diseases, such as SLE. Th17 cells produce inflammatory cytokines, such as IL-17A, IL-17F, and IL-23, which further exacerbate diseases and damage multiple organs (Paquissi and Abensur, 2021). Retinoic acid receptor–related orphan receptor γt is a key factor regulating the development and secretion of Th17 cells. The reduction of retinoic acid receptor–related orphan receptor γt effectively alleviates the symptoms of various autoimmune diseases. Three types of loquat leaf methyl coralline, ursodiol, oleanolic acid, and acetyl oxyl-oleanolic acid, isolated from *Osmanthus* were found to be effective inhibitors of Th17 differentiation and IL-17A secretion, which further reduces serum anti-dsDNA antibody levels, renal pathological damage, and antibody complex accumulation, improving the pathological damage and reducing the severity of renal damage in LN (Zhou et al., 2020).

### Realgar

**FIGURE 2:**
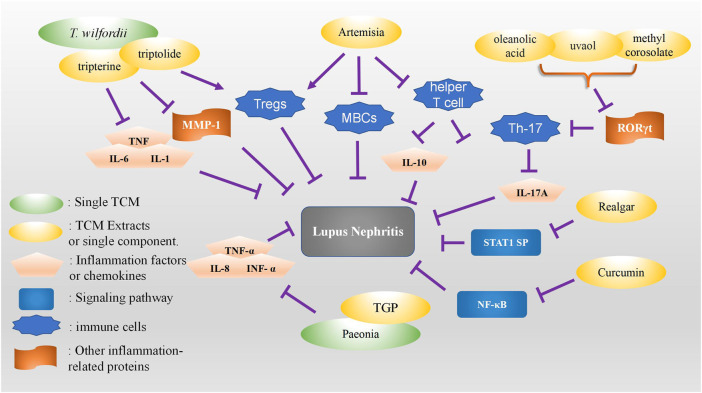
Summary of the role of TCM monomers and herbal or natural extracts in the treatment of lupus nephritis.

Realgar, an arsenic tetrasulfide compound, is a highly recognized TCM prescription that has been extensively used to treat various diseases, such as inflammatory diseases. However, in clinical treatment, high oral dose and high toxicity potential of realgar remain a problem. A previous study evaluated the effect of realgar nanoparticles on LN in MRL/lpr mice. The results showed that nano-realgar may be a potential agent for LN treatment, and it down- regulated the expression of p-STAT1, suggesting that nano-realgar may be one of the therapeutic targets of LN (Xu et al., 2019) (Figure 2).

## Conclusion

LN, by reason of its complex pathogenesis, remains as a therapeutically challenging chronic disease. Because of the increasing number of clinical research studies, more drugs and therapeutic regimens have been adopted in the clinical treatment of LN. In the long-term application of Western medicine, drug resistance gradually develops, and therapeutic efficacy subsequently declines. Despite the limited evidence on the efficacy of TCM for LN treatment, the aforementioned data suggest that TCM and Western medicines may have a synergistic effect. Their combination increases treatment efficacy, reduces toxicity and the disease recurrence rate, delays disease progression, and decreases the adverse effects, which suggest that this approach is worthy of promotion and application in clinical practice. Hence, more multicenter, large-sample double-blind randomized controlled trials and related basic experiments are necessary to further verify the mechanism of action, efficacy, and safety of integrated TCM and Western medicine in LN treatment and to formulate effective individualized treatment strategies. Accurate and reasonable understanding of the toxicity of TCM and its extracts is also necessary to provide a broader prospect for TCM in LN treatment clinically.
